# Preliminary analysis of reproductive, behavioral and physiological characteristics of military working dogs

**DOI:** 10.1590/1984-3143-AR2021-0092

**Published:** 2022-02-21

**Authors:** Graziele Braido Arcuri, Messy Hannear de Andrade Pantoja, Cristiane Gonçalves Titto, Daniele dos Santos Martins

**Affiliations:** 1 Departamento de Medicina Veterinária, Faculdade de Zootecnia e Engenharia de Alimentos, Universidade de São Paulo, Pirassununga, SP, Brasil; 2 Departamento de Zootecnia, Faculdade de Zootecnia e Engenharia de Alimentos, Universidade de São Paulo, Pirassununga, SP, Brasil

**Keywords:** behavior, semen quality, military dog, breed, welfare

## Abstract

The use of dogs in military work environments has always aroused great interest in the general population and determining the stress levels they go through is extremely important to maintain their welfare. The aim of this research was to evaluate if the work shifts in military working dogs leads to stress conditions and if this working influences on the reproductive performance and life quality. The study was conducted at the Military Police Kennel located at Campinas, Sao Paulo, Brazil. Eight male dogs of four different breeds (German Shepherd, Belgian Malinois Shepherd, Doberman, and Rottweiler) were evaluated during two different shifts: Working Shifts: animals working 12 hours a day with 2 hour-interval; and Control Shifts: animals that were on their day off (36 hours). Saliva samples were collected for cortisol analysis at the control and working shifts. The study was carried out over 60 days and analyzed behavior, physiology, and reproduction quality. Saliva samples, behavior observation of stereotyping, resting and moving activities and semen analysis were collected by digital stimulation (for combined second and third fractions). The salivary cortisol levels during the control and working shifts were between 0.361–0.438 and 0.312–0.592 µg/dL, respectively; the highest values were found at the end of working shifts. The animals were resting during most of the observation period, but few showed stereotypic behaviors. The testicular consistency was firm and semen parameters were within the normal values in German Shepherd, Belgian Malinois Shepherd, and Doberman dogs. However, Rottweiler dogs had a higher rate of sperm abnormalities, higher salivary cortisol levels, and more stereotypic behaviors. Nevertheless this work highlights the importance of further research relating reproduction and cortisol levels in military dogs.

## Introduction

Dogs have been active members of society since old times and they act to protect and provide company, including human work functions (Broom and Fraser, 2010). Specialized trained dogs work in forensic sciences to help on crime solutions, through their keen sense of smell, as they can identify explosive, drugs, chemical substances, or even human individual odor (Burghardt, 2003; Haverbeke et al., 2008). Actually, the demand for canines highly capable explosives detection due to the increases in emerging sophisticated threats needs (Lazarowski et al., 2020). Using dogs for working functions requires a different intensity of effort in comparison to dogs designed as pets, to expositions or laboratory activities (Möstl and Palme, 2002; Rooney et al., 2016).

Therefore, these animals may be more susceptible to stress, which leads to negative behavioral, hormonal (Hekman et al., 2014; Petersson et al., 2017), and reproductive changes (Kolster, 2018; Kumar et al., 2019). Impaired welfare results in abnormal behaviors, including running toward or away from the owners, hypervigilance (Mills et al., 2014), circling repeated turning, licking of objects for extended periods with no obvious purpose, or fixation to an item (Hall et al., 2015). Therefore, under stressful conditions, the behavior and cortisol levels of dogs should be evaluated because the plasma cortisol levels increase in response to stress (Starling et al., 2005; Chmelíková et al., 2020).

In addition to inducing behavioral and hormonal changes, stress can significantly decrease the quality of semen, such as decrease in sperm motility, increase in the number of dead sperm, increase in number of spermatic membrane alterations, high cytoplasmic drop retentions, and acrosomes alterations, indicating an impaired function of the epididymis (Baptista et al., 2009).

The knowledge of acceptable welfare patterns to reduce stress, which impairs the quality of life and fertility in dogs, can improve the management efficiency of working dogs. Besides a better performance of these military working animals, it is an important contribution to police, public safety, and crime preventive areas.

The study aimed to evaluate the possible effects of stress on the management of military working dogs by evaluating their behavior, salivary cortisol level, and semen parameters.

## Methods

### Animals and facilities

The animal study was reviewed and approved by the Ethics Committee of the Veterinary Medicine School at the University of São Paulo, Brazil (protocol number: 2633191120). Written informed consent was obtained from the owners for the participation of their animals in this study

The experiment was conducted at the 1^st^ Especial Action Police Crowd (in Portuguese BAEP), Campinas, SP, Brazil. Eight male dogs (weight: 40 kg, age: 48 months, work experience: 36 months) of four different breeds two German Shepherd (weight: 28 and 31kg, age: 4 and 3 years old respectively), two Rottweiler (weight: 38 and 40kg, age: 2 and 3 years old respectively), two Doberman (weight: 40 and 43kg, age: 3 years old) and two Belgian Shepherd Malinois (weight: 25 and 27kg, age: 5 and 4 years old respectively) were used.

The animals were housed in individual kennels (dimensions H × L × W: 2.1 × 4 × 2 m), with 3 m^2^ of roofing area and 5 m^2^ of a solarium. All houses had a water hole with potable water and a metal feeder, sewage pipe, wooden floor (2.25 m^2^), door with glass window and security locks, cemented floor with gradual inclination toward the drain and artificial lights according to the recommendations of the Decree 40400, October 24 1995 (São Paulo, 1995). The animals were fed commercial premium meals once a day, according to the National Research Council requirements, and had *ad libitum* access to the potable water. The dogs are vaccinated and dewormed according to the kennel calendar. In addition, clinical examination (complete physical examination) and laboratory analysis (blood and urine) were performed periodically.

All animals started basic training 6 to 8 months and extended to 12 months. All animals have the same training and after 12 months the animal starts its field work. From 12 months the animals continue in constant training to reinforce the knowledge acquired. Military dogs must be trained until the end of their military career, but always keeping the same objective. Each animal has a trainer, a policeman who takes care of the dog on working days. When the trainer has an off day, the animal remains in its dog house. On off days, all animals are cleaned and receive food and water. All dogs have a 12h working routine. The dogs undergo military training from 7:00 a.m. to 10:00 a.m. The working routine starts at 6:00 a.m. until 10:00 a.m. and restarts at 1:00 p.m. until 6:00 p.m. when the dogs go back to their kennel. After one work day of 12 hours, the animals rest for 36 hours.

### Experimental design

The experiment was divided into two different shifts: control shift (C) when animals day-off working and the working shift (W) when animals were working in military routine. The working military daily routine involves obey basis, military physical conditioning and sniffing (to drugs, people, firearms or other weapons). The military work includes street policing, where the dog goes to the police car and is engaged with narcotics occurrences, criminal and weapons finding.

There was an adaptation period (7 days) to the management procedures, subsequently the experiment was carried out during 60 days for evaluations of behavior, hormonal and reproductive analysis.

### Evaluation of animal behavior

Animal behavior was performed individually inside their kennels, by visual and direct observations, with instantaneous recording by focal sampling, from 6:00 a.m. to 8:00 a.m., and from 4:00 p.m. to 6:00 p.m., five minutes duration to each dog in each period. The total time to each animal was 600 seconds. The behavioral variables observed were (Haverbeke et al., 2008): Stereotypic behaviors, rest and movement behaviors (Table 1).

**Table 1:** Behavioral description (adapted Haverbeke et al., 2008).

***STEREOTYPIC BEHAVIOR***	
Repetitive walking	Repeat immediately a path just taken and continuing in the repetition; in circles, in a figure in the form of eight or walking in line on the fence / wall
Circling	Continuous walking in short circles, apparently chasing its tail or hind limbs
Manipulation of environment	Stereotypic interactions with elements from the environment; digging (= scratching the floor with the forepaws to a way that is similar to when dogs are digging holes); floor licking (= licking the floor with the tongue); rubbing legs against bars, gnawing at bars or at other material of the environment
***REST BEHAVIOR***	
Prone, head up	Trunk of body on cage floor
Sit	Hindquarters and front paws only in contact with cage floor
Sit, head in the grid	Hindquarters and front paws only in contact with cage floor. head in contact with the grid of cage
Stand	Upright with at least three paws in contact with cage floor
Oral behaviors	Barking, roaring, growling, whining, yelping
Prone, head down	Trunk of body on cage floor, chin or side of head in contact with cage side or floor, paws or limbs
Stand on two legs	Upright with at least two paws in contact with floor
***MOVEMENT BEHAVIOR***	
Walk	Takes at least one step, shifting body position
Sniff	Nose moved along objects and/or clear sniffing movements are exhibited
Urinating	Urinating while raising one hind limb posteriors
Defecating	Excreting the contents of the bowels
Drinking	Drink water from the drinking fountain
Oral behaviors	Barking, roaring, growling, whining, yelping
Eating	Eating food from the feeder

### Cortisol levels

During the WS, the saliva samples from each dog were collected four times a day for 3 days—T0: before training (7:00 a.m.), T1: after training (10:00 a.m.), T2: before training after a rest period (1:00 p.m.), and T3: at the end of the daily routine (7:00 p.m.). During the CS, the saliva samples from each dog were collected two times a day for 3 days—morning (7:00 a.m.) and afternoon (6:00 p.m.).

For determination to cortisol concentrations, saliva samples were collected by using the Salivette^®^ tubes (SARSTEDT Ref 51.1534.500) that have a high absorbable swab. The oral cavity of the animals was previously cleaned with filtered water and then the animals were stimulated to chew the swab for three to five minutes.

The saliva samples were centrifuged at 2,000 rpm for 5 min and the supernatant was separated and stored at -20ºC. Subsequently, the cortisol level was quantified using an electrochemiluminescence method. The mean intra- and inter-assay coefficients of variation were 7.1% and 11.5%, respectively.

### Semen quality

The Testicular consistency was measured by palpation, always by the same technician. Using a scale from one to five for classification, where: 1- friable consistency; 2- intermediate consistency; 3- firm consistency (desirable); 4- turgid consistency and 5- hard consistency (Baptista et al., 2009).

The semen was collected in both experimental periods by digital manipulation (Kutzler, 2005) for combined second and third fractions and maintained at 37°C. After collection, the proportion of motility spermatozoa present in the semen samples was immediately estimated subjectively using optical microscopy (100x) (CBRA, 2013) and referred as overall sperm motility. A semen sample was thereafter in buffered formol-saline solution could be used to detect nuclear, acrosome and tail abnormalities, as well as the location of cytoplasmic droplets, on 200 spermatozoa per sample

The spermatic concentration (CONC×10^6^ sptz/mL) was evaluated in a Neubauer chamber. The semen was diluted 1:20 a 50-μL aliquot of ejaculated and 950μL of distilled water or 10% buffered saline formalin. The sperm count was performed under phase contrast microscopy, with a magnification of 1000x (Ax et al., 2004).

The sperm morphology was performed by semen smear, using glass slides, fixed with saline formalin during 10 min at water bath 37 ºC, dried at room temperature and stored. The slides were colored by the Karras modified method (Papa et al., 1988), and counted 200 cells, using phase contrast optical microscopy under magnification of 1.000x. The spermatozoa were evaluated and classified as minor sperm defects (MiD, %, head defects, tail defects and implantation, and distal cytoplasmic droplets), major defects (MaD, %, acrosome defects, head defects, proximal cytoplasmic droplets, teratological forms, abnormal midpiece, tail defects and double forms) and total defects (TD, %) (Ax et al., 2004).

### Statistical analysis

The salivary cortisol level data were analyzed by variance analysis (MIXED-SAS) using two approaches—1) to compare the WS and CS, fixed effects of breed, time (7 a.m. and 6 p.m.), the shifts, and its current interactions were used; 2) to evaluate only the WS, fixed effects of breed, time (7 a.m., 10 a.m., 1 p.m., and 6 p.m.), and its interactions were used. Animals were used as repeated measures. The average results were compared using the F test, T test, or Tukey–Kramer test as applicable.

Behavior data were analyzed using exploratory analysis, with the objective of characterizing data distribution and the source of relevant variations, by those results, adjusted model, and general linear theory models (GLIMMIX-SAS). To evaluate each behavioral variable, starting on the percentage of frequencies of different occurrences of variable categories related to the behavioral variables, the data were adjusted to “Arc-Sen percentage root” and then, variance analysis was performed. Statistics model considered as fixed effects breed, day period (morning or evening), shifts (working or control), and its interactions to multiple comparisons procedure (PDIFF). Animals were used as repeated measures. To present the results, the data were turned back to the original shifts. All tests were performed at 5% probability. SAS 9.3 software (2012) was used for all analyses.

## Results

### Animal behavior

The behavior analysis evidenced resting phases during most of the time (Table 2).

**Table 2:** Mean values of the daily (morning and afternoon) behavior of military working dogs of different breeds expressed as seconds per activity.

**Behavior**	**Breeds**				**P value**
	**German Shepherd**	**Belgian Malinois**	**Doberman**	**Rottweiler**	
**Stereotypic Behavior**					
Repetitive walking	3.95 b	2.45 b	0 b	26.0 a	0.005
Circling	0	0	0	2.30	0.397
Manipulation of environment	0	0	0	0.54	0.397
**Rest behavior**					
Prone, head up	111.75 a	141.21 a	162.42 a	78.29 b	0.050
Sit	54.00 a	44.08 ab	48.42 a	34.37 b	0.036
Sit, head in the grid	49.29 a	8.75 b	19.67 b	9.41 b	0.025
Stand	26.67 b	76.33 a	19.42 b	60.79 a	0.004
Oral behaviors	4.67	1.67	3.42	2.54	0.515
Prone, head down	25.00	0	22.00	37.50	0.376
Stand on two legs	5.87	0	6.83	0.33	0.415
**Movement behaviors**					
Walk	8.29 b	25.04 a	11.62 b	30.21 a	0.021
Sniff	0.62	0	1.54	0.21	0.679
Urinating	0	0	0	0	0
Defecating	0	3.62	0	2.29	0.548
Drinking	4.75	4.83	1.29	6.46	0.331
Oral behaviors	2.87	3.33	1.71	2.62	0.956
Eating	2.25	0	0	7.79	0.412

a,b different lowercase letters in the rows (inside category) indicate significant difference (p < 0.05). A total time of 300 seconds of observation for each breed.

Prone, head up, the most observed behavior (*P* < 0.05), did not vary among the breeds (*P* > 0.05). Sit and sit and head in the grid were the most observed behaviors in German Shepherd (*P* < 0.05) dogs, the stand was the most observed behavior in Belgian Shepherd Malinois and Rottweiler dogs. German Shepherd dogs showed greater oral behaviors (*P* < 0.05) than other dogs. Standing on two legs was the most observed behavior in German Shepherd and Doberman dogs (Table 2).

Regarding movement behaviors, the most observed behavior was walk in Rottweiler dogs and Belgian Shepherd Malinois dogs compared to that in other dogs, while sniffing, defecating, drinking, oral behaviors, and eating did not show significant differences among the breeds (*P* > 0.05). Urinating was not observed during the entire period.

Stereotypic behaviors were higher in Rottweiler dogs (*P* < 0.05) than German Shepherd, Belgian Malinois Shepherd, and Doberman dogs.Doberman dogs did not show stereotypic behaviors (Table 2).

Comparing the WS and CS during the periods (morning and afternoon), stereotypic behaviors were high in the morning on both shifts (*P* < 0.05), and they were not observed in the afternoon. The resting behavior was longer in the afternoon in both shifts, without significant differences, while it was significantly higher in the morning in the CS than in the WS (*P* < 0.05; Table 3).

**Table 3:** Mean values of behavior of military working dogs of different breeds during the working and control shifts in the morning and afternoon expressed as seconds per activity.

**Behavior**	**Working shifts (WS)**		**Control shifts (CS)**		**P value**
**Morning**	**Afternoon**	**Morning**	**Afternoon**
**Stereotypic behaviors**					
Repetitive walking	25.2 a	0 b	7.2 b	0 b	0.0186
Circling	0	0	2.33	0	0.320
Manipulation of environment	0	0	0.54	0	0.320
**Rest behaviors**					
Prone, head up	49.37 c	165.42 a	110.75 b	168.13 a	0.018
Sit	72.84 a	44.91 b	22.04 b	41.08 b	0.001
Sit, head in the grid	28 b	10.5 c	44 a	4.62 c	0.067
Stand	77.08 a	23.87 c	54.29 b	27.97 c	0.195
Oral behaviors	5.2 a	0 c	5 a	2.08 b	0.701
Prone, head down	5.17 b	25 a	25 a	29.34 a	0.612
Stand on two legs	7.17 a	0 b	5.87 b	0 b	0.309
**Movement behaviors**					
Walk	22.34	12.7	25.75	14.37	0.079
Sniff	0	0	0	2.37	0.103
Urinating	0	0	0	0	0
Defecating	0	5.91	0	0	0.160
Drinking	1.41	9.17	1.37	5.37	0.177
Oral behaviors	6.5	2.25	0	1.79	0.070
Eating	0	0	0	10.04	0.309

a,b different lowercase letters indicate significant difference in the rows (p < 0.05). A total time of 300 seconds of observation for each period (morning/afternoon).

### Cortisol levels

**Figure 1:**
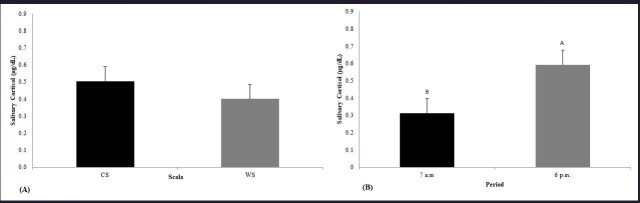
Mean values (±standard error) of salivary cortisol measured working and control shifts (A) and Salivary Cortisol measured at 7 a.m and 6 p.m in the working and control shifts (B). Different capital letters (A, B) indicate significant difference within the periods or time (p < 0.05).

The salivary cortisol level in the WS was 0.504 µg/dL and did not differ from that in the CS (0.400 µg/dL; Figure 1A). However, differences between periods were observed; the higher average level was detected at 6 p.m. than at 7 a.m. (0.592 µg/dL vs. 0.312 µg/dL; *P* = 0.0234; Figure 1B). No interactions between the shifts and periods were observed (*P* > 0.05).

**Figure 2:**
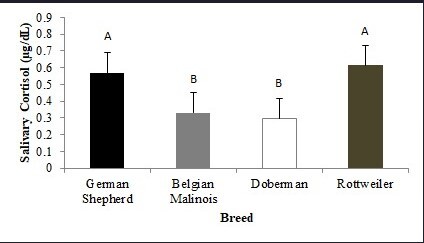
Mean values (±standard error) of salivary cortisol of German Shepherd, Belgian Shepherd Malinois, Doberman and Rottweiler measured at 07 a.m and 6 p.m. A, B different capital lowercase letters indicate significant difference in the rows (p < 0.05).

**Figure 3:**
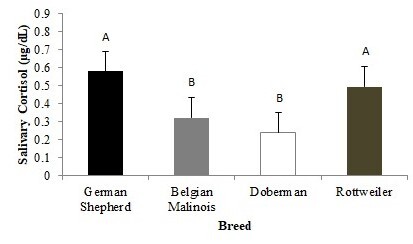
Mean values (±standard error) of salivary cortisol during the working shifts at different breeds. A,B different capital lowercase letters indicate significant difference in the rows (p < 0.05).

Rottweiler (0.614 µg/dL) and German Shepherd (0.567 µg/dL) dogs had higher salivary cortisol levels (*P* > 0.05) than other dogs (Figure 2) at 7 a.m. and 6 p.m. In the WS, German Shepherd and Rottweiler dogs had higher (*P* < 0.05) salivary cortisol levels than Doberman and Belgian Shepherd Malinois dogs (Figure 3).

**Figure 4:**
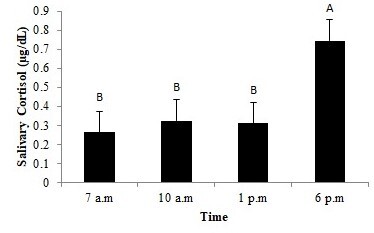
Mean values (±standard error) of salivary cortisol of military dogs during working shifts. A,B different capital lowercase letters indicate significant difference in the rows (p < 0.05).

When considering the different periods in the WS, there were no differences in the levels (*P* > 0.05) among the samples at 07:00 a.m. (0.264 µg/dL), 10:00 a.m. (0.324 µg/dL), and 1:00 p.m. (0.312 µg/dL); however, the levels were higher (*P* < 0.05) at 6:00 p.m. (0.745 µg/dL; Figure 4).

### Semen quality

All testicles were in firm condition as desired. Semen analysis was according to the pattern for dogs and was based on a total of eight samples (Supplementary Material 1). German Shepherd, Belgian Malinois Shepherd, and Doberman dogs had PM values between 80% to 90%, while Rottweiler dogs had PM values of 60% (Table 4). German Shepherd, Belgian Malinois Shepherd, and Doberman dogs had VIG values between 3 and 4 (good), while Rottweiler dogs had VIG values of 2 (very slow).

**Table 4:** Values of spermatic concentration (CONC), sperm vigor (VIG), progressive sperm motility (PMot), minor defects (MiD), major defects (MaD), and total defects (TD) of military working dogs.

**Breed**	***CONC***	***VIG***	***PMot***	**MiD**	**MaD**	**TD**
***(×10^3^ sperm/mL)***	***(0–5)***	***(%)***	***(%)***	***(%)***	***(%)***
German Shepherd	128.356	3	80	2.5	10.5	13.0
German Shepherd	110.544	3	80	3.0	5.5	8.5
Belgian Malinois	136.128	4	90	2.0	6.5	8.5
Belgian Malinois	129.124	4	90	2.5	7.5	10.0
Doberman	126.020	3	80	4.0	8.0	12.0
Doberman	131.226	3	80	3.0	5.0	8.0
Rottweiler	18.992	2	60	4.0	26.5	30.5
Rottweiler	20.826	2	60	3.0	25.0	28.0

The sperm concentration varied between 100,000 and 136,000× 10^3^ sperm per mL for German Shepherd, Belgian Malinois Shepherd, and Doberman dogs and between 18,000 and 20,000 × 10^6^ sperm per mL for Rottweiler dogs. Minor and major defects, considering different pathologies, were found in all breeds, and a higher number of defects were found in Rottweiler dogs (total defects +-29,25%) than in other dogs.

## Discussion

Behavior characteristics are related to different physiological and neuroendocrine responses under stress (Etim et al., 2013). Similar to our study, when the military dogs finished their work day they exhibited stereotypic behaviors such as repetitive walking behavior and Oral behaviors, which can be a sign of restlessness (Foyer et al., 2016). This restlessness can be attributed to previously defined situations as stimuli that could be anticipated (e.g. training method) or the presence of a human (Beerda et al., 1998). Increased Oral behaviors may be associated with training, as dogs showed more this behavior when training together (Clark et al., 1997).

During the resting period, prone, head up was the most frequent behavior for all breeds; however, there was no apathy or periods of inactivity, which is in contrast to that observed in challenged dogs (Beerda et al., 1997; Haverbeke et al., 2008), shelter dogs (Hubrecht et al., 1992), and laboratory dog models (Hetts et al., 1992), which were under similar conditions. The dogs were relaxed when sitting down or laying down with no tension in muscles (Lindsay, 2005), similar to our study.

No animal showed stereotypic behaviors like to had excessive corporal manipulation (scratching, licking, and biting), coprophagy, excessive vocalization, leg lifting; repeated sniffing and urination, and stereotypic behaviors (Hubrecht et al., 1992; Hetts et al., 1992; Beerda et al., 1997; Haverbeke al., 2008).

The similarities in the animal’s growth and their given goals since their puppy phase in the same kennel system (Hennessy et al., 1998; Stephen and Ledger, 2005) can explain the lower variation in all behaviors assessed between breeds. Rottweiler dogs had different behaviors from other dogs, which can be explained by the individual perception of the environment. The individuality among the breeds can be related to the temperament and motivation to associate specific activities (Schilder, 1992; Coppinger and Schneider, 1995). Furthermore, since Rottweiler dogs show energetic behavior, it is essential that these dogs have enough space for constant physical exercises; otherwise, they may experience stress and their behavior may be affected mainly by limited physical activity or due to the insufficient amount of training to dispense energy (Beauchamp, 2000; AKC, 2020).

The basal salivary cortisol level in dogs ranges from 0.02 to 0.3 μg/dL (Bennett and Hayssen, 2010; Wenger-Riggenbach et al., 2010). In this study, the average salivary cortisol level in the CS was between 0.361 and 0.438 μg/dL and that in the WS was between 0.312 and 0.592 μg/dL. This result implies that even under resting conditions (CS), working dogs have high cortisol levels, which means they can be stressed for a long period of time (Hekman et al., 2012).

The cortisol level during the WS did not show significant alterations, probably because the animals were under the same stimuli, showing that they are able to deal with the same challenges since their training routine and the environment were the same for all the breeds, with small or no variations in cortisol levels (Haverbeke al., 2008). An increase in cortisol levels occurred when the dogs returned from work due to external activities. They encounter constant challenges because they are exposed to various uncommon situations such as traffic jams, distraction such as noises, and unknown people and animals (Svobodova et al., 2014). Circadian rhythm of cortisol increases at night and decreases during the day, which is similar in clinically normal dogs (Camacho, 1987). Also, normalization of the cortisol levels always occurred within 60 min following the administration of one unique stimulus (unpredictable shock) in dogs (Beerda et al., 1998), and the peak of cortisol levels can occur between 60 to 120 minutes after a punctual stress (Henrique et al., 2017).

Behavior characteristics are related to different physiological and neuroendocrine responses under stress and are consistent. Some studies have found only physiological or behavioral differences (Beerda et al., 1997), similar to our study, when the dogs finished their work day. Occasionally, when the cortisol level is higher and there are no behavioral activities related to stress, cortisol levels may be correlated to physical work (Sundman et al., 2019). This was observed for German Shepherd dogs, which showed high cortisol levels, but their behavior was not affected (Foyer et al., 2016). However, Rottweiler dogs had higher salivary cortisol levels than other dogs, possibly because these dogs exhibited greater stereotypic behaviors such as repetitive walking behavior and movement behavior (walk), which can be a sign of restlessness.

Our findings show that it is possible that Rottweiler dogs are more susceptible to stress as they present a different response than other dogs subjected to the same stimuli during training, rest, or military work. Dogs have different coping strategies responses to the same stimulus (Chmelíková et al., 2020) due to several reasons such as breed, temperament, personality, and previous individual experiences (Marliani et al., 2018). The breed has a great effect on the personality of the animal and, consequently, on the suitability of the dog to the military working (Netto and Planta, 1997).

The breed factor is also important in the analysis of dog semen, since total sperm in the ejaculate is based on testicular size and there is also a shortage of specific information of the breed (Kustritz, 2007). In semen analysis, the German Shepherd, Belgian Malinois Shepherd and Doberman, and dogs showed satisfactory results for all the reproductive parameters. The Rottweiler dogs semen analysis showed lower sperm count, PM, VIG, MiDs, MaDs, and TDs. As previously described in the Rottweiler breed, negative changes in behavior can be related to stress. The response to stress activates the hypothalamic-pituitary-adrenal axis and releases cortisol that possibly affected semen quality (Curley et al., 2008). High glucocorticoid levels decrease the production and secretion of testosterone from the Leydig cells and spermatogenesis (Brownlee et al., 2005), a process of division and differentiation of germ cells into spermatozoa (Johnson et al., 2000). Any change in this process has a negative effect on the semen quality such as sperm count, percentage of motile sperm, and percentage of morphologically normal sperm, which is observed in the Rottweiler breed (Janevic et al., 2014; Faeza et al., 2019). The low percentage of normal sperm morphology exhibited by the Rottweiler breed is possibly due to the malfunction of the epididymis because of the influence of stressors (Almeida et al., 1998). Stress factors also significantly increase MaDs, which can be associated with low fertility semen, but these abnormalities cannot be related to infertility (Vannucchi et al., 1998; Kumar and Singh, 2015). Infertility may be due to a combination of low spermatic concentration, poor sperm motility, or abnormal sperm morphology (Kumar and Singh, 2015).

Despite having higher TDs, the fertility of the Rottweiler dog is not affected as >69.5% sperm are normal and fertility can be affected when <60% of the sperm are abnormal (Oettlé, 1993). In addition, other factors must be considered regarding the low performance of the Rottweiler dogs as the breed factor is important in the analysis of the semen from dogs, the total sperm count in the ejaculate is based on testicular size and there is also a shortage of specific information of the breed (Kustritz, 2007).

The semen parameters are positively correlated with each other, the PM is positively correlated with spermatic concentration (Agarwal et al., 2003) and the higher percentage of abnormal sperm is positively correlated with PM in dogs (Ellington et al., 1993); therefore, the number of sperm pathologies found in the Rottweiler breed may have contributed to the lower PM values than that indicated for the species, which is around 70% (Freshman, 2002), and VIG below three (CBRA, 2013). These findings are similar to those described in Rottweiler working dogs, which also had the same modification patterns, except for spermatic concentration and minor defects (Baptista et al., 2009).

## Conclusion

In this study we conclude that military dogs presented more stressful signs when they are in working period as stereotypic behavior, but cortisol levels and semen quality was not affected, only when breeds were analyzed separately. However, as we have a small sample size of each breed, results must be analyzed carefully.

## Supplementary Material

Supplementary material accompanies this paper. Supplementary Material 1 - Semen characteristics

This material is available as part of the online article from https://www.scielo.br/j/ar
