# Supplementary material for: Preliminary analysis of reproductive, behavioral and physiological characteristics of military working dogs
Source: Anim Reprod. 2022 Feb 21;v19(1):e20210092. doi: 10.1590/1984-3143-AR2021-0092 (PMC8893309; doi:10.1590/1984-3143-AR2021-0092)
Supplement: Supplementary material accompanies this paper. [file 1984-3143-ar-v19-1-e20210092-suppl1.pdf]

Supplementary Material 1- Semen characteristics

ANIMAL	***BREED***	***VOLUME (ml)***	***COLOR***	***SMELL***	***Ph***	***MOTILITY - (%)***	***VIGOR (0-5)***
Kelvin	Belgian Shepherd Malinois	6.5	white	*sui generis*	6.2	80	3
Gringo	Belgian Shepherd Malinois	7	white	*sui generis*	6.2	90	4
Egar	Rottweiler	8.5	translucent	*sui generis*	6.6	60	2
Thor	Rottweiler	7.8	translucent	*sui generis*	6.5	60	2
Pegus	German Shepherd	7.5	white	*sui generis*	6.2	80	3
Falcon	German Shepherd	7	white	*sui generis*	6.2	90	4
Lotan	Doberman	6.5	white	*sui generis*	6.3	80	3
Connors	Doberman	7	white	*sui generis*	6.4	80	3

Fonte: ARCURI, G.B. (2015)
